# Biomarker conversion from primary breast cancer to synchronous axillary lymph node metastasis and neoadjuvant therapy response: a single-center analysis

**DOI:** 10.1007/s00432-024-05834-y

**Published:** 2024-06-08

**Authors:** Mao Ding, Mengyuan Li, Qian Liu, Ling Xu

**Affiliations:** Department of Thyroid and Breast Surgery, Peking University First Hospital, No.8 Xishiku Street, Beijing, 100034 China

**Keywords:** Breast cancer, Lymph node metastasis, Receptor, Discordance, Neoadjuvant therapy

## Abstract

**Purpose:**

The biomarker characteristics of breast cancer plays an important role in predicting treatment sensitivity. The aim of the present study was to compare immunohistochemical profiles (ER, PR, HER2, and Ki67) between the primary tumor and synchronous axillary lymph node metastasis and investigate the subsequent effects on neoadjuvant therapy response.

**Methods:**

A total of 358 patients with pathologically confirmed synchronous axillary lymph node metastasis at first diagnosis and treated by neoadjuvant therapy at Peking University First Hospital from January 1, 2013 to December 31, 2022 were included in this retrospective study. Clinicopathologic data, especially receptor status in primary and metastatic foci, was collected for each case.

**Results:**

Change of ER, PR, HER2, and Ki67 expression was observed in 5.9%, 8.7%, 12.6%, and 17.3% of patients, respectively. HR discordance was observed more frequently when the ER status (*p* = 0.023) or PR status (*p* = 0.010) of primary tumor was negative, while HER2 discordance seemed to be more frequent when the HER2 status of primary tumor was HER2-0 or HER2-low (*p* < 0.001). Patients with loss of HR-positivity (positive to negative) responded to neoadjuvant chemotherapy better compared to those with stable positive HR expression (50% vs. 11.1%, *p* = 0.0017). A significantly decrease in pCR rate was observed in patients with unstable HER2 status, but not in the HER2-0/HER2-low subgroup.

**Conclusion:**

Receptor discordance between primary tumor and synchronous axillary LNM appears to already exist before any anti-tumor therapy. This instability has limited clinical impact on the choice of neoadjuvant therapy at current stage, but further investigation is warranted with the incremental application of endocrine drugs and ADCs in neoadjuvant therapy.

**Supplementary Information:**

The online version contains supplementary material available at 10.1007/s00432-024-05834-y.

## Introduction

Breast cancer is recognized as a highly heterogeneous disease that accounts for almost one third (31%) of female cancers (Siegel et al. 2023). In the past decades, the field of breast cancer treatment has gradually entered the era of precision medicine. Oncologists now classify invasive breast cancer based on immunohistochemical analysis of estrogen receptor (ER), progesterone receptor (PR), human epidermal growth factor receptor 2 (HER2), and proliferation factor Ki67, which not only provides prognostic information but it is also crucial for predicting treatment sensitivity. However, previous studies have reported changes in immunohistochemical expression of these four biomarkers between primary and metastatic tumors, and there may be even a significant prognostic impact of phenotype conversion (Lindstrom et al. 2012; Mellouli et al. 2022). Discordance in these tumor biomarkers has been reported due to analytical error, intratumoral heterogeneity, selective pressure of therapy and cellular clonal evolution (Shiino et al. 2022). Thus, current guidelines recommend biopsy of the first site of metastasis for recurrent or stage IV disease in order to decide on the appropriate first-line systemic therapy (Gradishar et al. 2023; Rugo et al. 2016).

Although the current studies primarily focus on distant metastasis, several studies with small sample size have evaluated the discordance in biomarker status between primary tumors and synchronous axillary lymph node metastasis (LNM) based on surgical specimens, showing lower discordance rate (Falck et al. 2010; Janeva et al. 2023; Li et al. 2016; Weydandt et al. 2022). Actually, the importance of pretherapeutic receptor expression in guiding treatment is increasing, because neoadjuvant chemotherapy has become part of the standard treatment regimen for locally advanced disease. However, in routine clinical practice, management of early invasive breast cancer patients with synchronous LNM is frequently based on the biomarker status of the primary tumor, ignoring the immunohistochemical analysis of LNM. Moreover, the successful development of antibody–drug conjugates (ADCs) challenged the traditional evaluation criteria for HER2 expression (Modi et al. 2022). High instability of HER2-low-positive expression from primary breast cancer to relapse has been reported, but the implications of this alteration in neoadjuvant therapy remain uncertain (Miglietta et al. 2021).

In the present work, we evaluated the prevalence of discordance in biomarker status (ER, PR, HER2, and Ki67) between the primary tumor and synchronous axillary LNM, as well as the impact of such alterations on neoadjuvant therapy response.

## Method

### Patients

The clinicopathological data of early invasive breast cancer patients treated with neoadjuvant therapy from the database of the Breast Disease Center of Peking University First Hospital from January 1, 2013, to December 31, 2022, were retrospectively reviewed. The inclusion criteria were patients with pathologically confirmed synchronous axillary LNM at first diagnosis and treated with neoadjuvant therapy. All patients were diagnosed by core needle biopsy (CNB) of primary breast foci, and lymph nodes were evaluated by needle biopsy if clinically positive or by sentinel lymph node biopsy (SLNB) if clinically negative or needle biopsy negative. The exclusion criteria were as follows: (1) multifocal primary invasive breast cancer lesion with an inconsistent ER, PR, HER2 or Ki67 status; (2) no standardized neoadjuvant therapy or surgery; (3) occult breast cancer; (4) patients only undergoing neoadjuvant endocrine therapy; (5) unavailable ER, PR, HER2 or Ki67 status and inability to retest.

### Clinicopathologic data

Clinicopathologic data, including age, menopause status, family history, clinical stage, histological grade, histological type, ER status, PR status, HER2 status, Ki67 status, and anti-tumor treatment regimens, were recorded. Clinical stage was determined according to the American Joint Committee on Cancer (AJCC) stage system (8th edition) (The American College of Surgeons 2017), based on physical examination, mammography, or ultrasonography of the breast and regional nodal at the fist diagnosis.

Immunohistochemistry (IHC) for ER, PR, HER2, and Ki67 was performed for both primary tumor and synchronous axillary LNM using the same automated IHC stainers and antibodies (Supplementary Table 1). According to the 2020 American Society of Clinical Oncology and the College of American Pathologists (ASCO/CAP) guidelines (Allison et al. 2020), ER and PR status were classified as negative (lack of any ER/PR immunoreactivity or < 1% immunoreactivity in tumor cells, with a positive internal control) or positive (≥ 1% immunoreactivity in tumor cells). Hormone receptor (HR) status was considered positive in case of ER and/or PR positivity. The evaluation of HER2 status followed the HER2 diagnostic guidelines revised by ASCO/CAP in 2013 (Wolff et al. 2013). For equivocal IHC results (IHC score 2 +), fluorescence in situ hybridization (FISH) assays were performed to test for HER2 gene amplification. Cases were considered as HER2-positive in case of IHC score 3 + and/or HER2 gene amplification by FISH, HER2-low in case of IHC score 1 + or IHC score 2 + plus the absence of HER2 gene amplification, and HER2-0 in case of IHC score zero (Tarantino et al. 2020). Ki67 was evaluated by the percentage of tumor cell nuclei with positive immunostaining, and ≥ 30% was defined as high (Nielsen et al. 2021). Histological type and grade of primary breast cancer were distinguished based on the World Health Organization (WHO) classification of breast cancer and Nottingham grading by two dedicated breast pathologists.

### Neoadjuvant treatment regimens and evaluation criteria

All patients received at least 4 cycles of neoadjuvant treatment which was conducted with reference to the National Comprehensive Cancer Network (NCCN) guidelines or the Chinese Society of Clinical Oncology (CSCO) guidelines. The first choice of treatment regimen was based on taxanes and/or anthracyclines. A subset of HER2-positive patients received HER2-targeted therapy with the TCHP (docetaxel/albumin-bound paclitaxel + carboplatin + trastuzumab + pertuzumab) or TCH (docetaxel/albumin-bound paclitaxel + carboplatin + trastuzumab) regimens.

Early clinical response evaluation was performed after 2–4 treatment cycles in accordance with Response Evaluation Criteria in Solid Tumors (RECIST) 1.1 (Eisenhauer et al. 2009). Complete response (CR) and partial response (PR) were considered effective. All patients underwent local therapy involving either lumpectomy (breast-conserving surgery) or total mastectomy after completion of neoadjuvant treatment, and pathological response was evaluated using Miller-Payne grading system (Ogston et al. 2003). According to the recommendation of Collaborative Trials in Neoadjuvant Breast Cancer (CTNeoBC), pathological complete response (pCR) was defined as the absence of invasive carcinoma in both breast and axillary lymph nodes, irrespective of ductal carcinoma in situ (ypT0/TisypN0) (Cortazar et al. 2014).

### Statistical analysis

Measurement data were described using the median and interquartile range (IQR) values; count and ranked data were described using the number of cases and percentages. Differences of biomarker status between matched pairs of primary breast cancer and synchronous axillary LNM were evaluated using McNemar-Bowker test. Cohen kappa statistic was calculated to estimate the overall agreement of biomarker status between the primary tumor and LNM. Kappa-values > 0.8, between 0.6 and 0.8, between 0.4 and 0.6, between 0.2 and 0.4, and < 0.2 were classified as very good, good, moderate, fair and poor agreement, respectively. Pearson’s *χ*^*2*^ test or Fisher’s exact test was used for univariate analysis and factors with *p* < 0.05 were included in the multivariate analysis. Logistic regression was performed to calculated the odds ratio (OR) and corresponding 95% confidence interval (CI). All tests were two-sided and a criterion of *p* < 0.05 was used for significance. All statistical analysis were performed using R 4.3.2 (http://www.r-project.org).

## Results

### Baseline clinicopathological characteristics

**Fig. 1:**
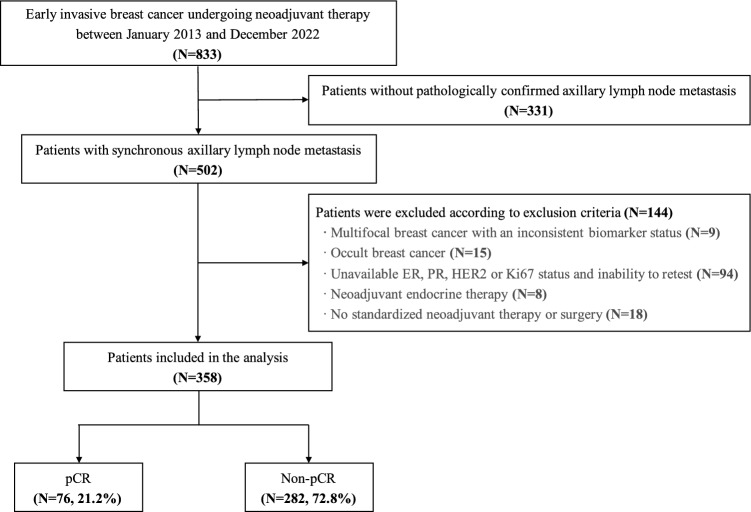
Flow diagram of the study. *ER* estrogen receptor, *PR* progesterone receptor, *HER2* human epidermal growth factor 2, *pCR* pathological complete response

A total of 833 patients with early invasive breast cancer received neoadjuvant therapy at Peking University First Hospital from January 1, 2013 to December 31, 2022. After the exclusion of ineligible cases, 358 cases with complete data were enrolled in this study (Fig. 1). All the patients were women. The median age of our cohort was 51 years (IQR 42.3–58.0) and 172 patients (48.0%) were postmenopausal when diagnosed with breast cancer. The majority of patients had tumors of ductal histology (*n* = 319, 89.1%) and poor differentiation (G3, *n* = 176, 49.2%) on baseline biopsy. There were 294 patients (82.1%) with primary tumor larger than 2.0 cm and 328 patients had clinical positive axillary lymph nodes. More than half of the cases (*n* = 197, 55.0%) underwent combination anthracycline/taxane based neoadjuvant chemotherapy and 146 patients (40.8%) received anti-HER2 blockade associated with neoadjuvant chemotherapy. Seventy-six patients achieved pCR after neoadjuvant therapy for a pCR rate of 21.2%. The baseline characteristics of the overall population were shown in Table 1.

**Table 1:** Characteristics of the patients at baseline

Characteristic	Patients (*N* = 358)
Age at diagnosis, years (median [IQR])	51 [42.25,58.0]
Sex	
Male	0 (0.0)
Female	358 (100.0)
BMI, kg/m^2^	
< 18.5	7 (2.0)
≥ 18.5, < 25.0	209 (58.4)
≥ 25.0, < 30.0	117 (32.7)
≥ 30.0	25 (7.0)
Menopausal status	
Pre/perimenopausal	186 (52.0)
Postmenopausal	172 (48.0)
Family history of breast cancer	
Yes	27 (7.5)
No	331 (92.5)
Laterality of primary tumor	
Right	172 (48.0)
Left	186 (52.0)
Histological type	
Invasive ductal	319 (89.1)
Invasive lobular	10 (2.8)
Other	29 (8.1)
Histological grade	
G1	12 (3.4)
G2	166 (46.4)
G3	176 (49.2)
Unknown	4 (1.1)
Clinical T stage	
cT1	64 (17.9)
cT2	237 (66.2)
cT3	51 (14.2)
cT4	6 (1.7)
Clinical N stage	
cN0	30 (8.4)
cN +	328 (91.6)
Biopsy method of axillary lymph node	
CNB	316 (88.3)
SLNB	42 (11.7)
Neoadjuvant chemotherapy	
Taxane based	160 (44.7)
Combination anthracycline/taxane based	197 (55.0)
Other	1 (0.3)
Neoadjuvant HER2-targeted therapy	
None	212 (59.2)
Trastuzumab alone	82 (22.9)
Trastuzumab plus pertuzumab	64 (17.9)
Early clinical response	
PD	12 (3.4)
SD	173 (48.3)
PR	149 (41.6)
CR	5 (1.4)
Unknow	19 (5.3)
Miller-Payne stage	
G1	52 (14.5)
G2	30 (8.4)
G3	109 (30.4)
G4	80 (22.3)
G5	84 (23.5)
Unknow	3 (0.8)
Pathological response	
pCR	76 (21.2)
Non-pCR	282 (78.8)
Breast surgery	
Mastectomy	277 (77.4)
Breast conserving surgery	81 (22.6)

*IQR* interquartile range, *BMI* body mass index, *CNB* core needle biopsy, *SLNB* sentinel lymph node biopsy, *HER2* human epidermal growth factor 2, *PD* progressive disease, *SD* stable disease, *PR* partial response, *CR* complete response, *pCR* pathological complete response

### Discordance in biomarker status between primary breast *cancer* and LNM

**Fig. 2:**
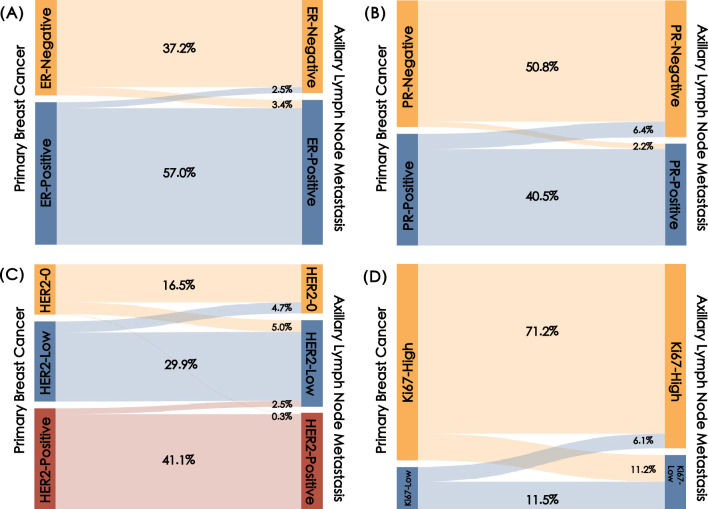
Biomarkers expression evolution from primary breast cancer to synchronous axillary LNM. **A** ER conversion. **B** PR conversion. **C** HER2 conversion. **D** Ki67 conversion. *ER* estrogen receptor, *PR* progesterone receptor, *HER2* human epidermal growth factor 2

**Fig. 3:**
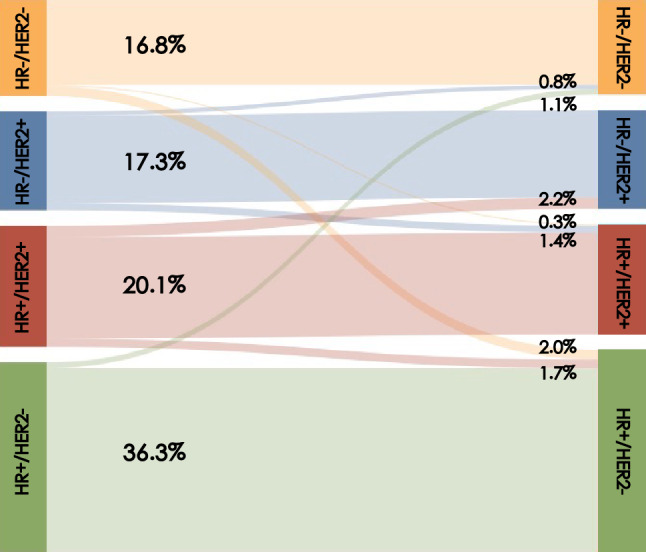
Subtype changes between the primary breast cancer and synchronous axillary LNM. *HR* hormone receptor, *HER2* human epidermal growth factor 2

The majority of the primary breast cancer and LNM were ER-Positive (*n* = 213, 59.5%), PR-Negative (*n* = 190, 53.1%) and HER2-Positive (*n* = 156, 43.6%), and so it is with LNM (Table 2). A receptor change occurred either as a receptor gain or as a receptor loss from primary tumor to metastasis (Fig. 2). Discordance in ER status was observed in 21 patients (5.9%, *p* = 0.663), with ER-gain conversion in 12 patients (3.4%) and ER-loss conversion in 9 patients (2.5%). PR status conversion was detected in 31 of cases (8.7%, *p* = 0.012), with PR-gain conversion in 8 patients (2.2%) and PR-loss conversion in 23 patients (6.4%). The overall discordance rate of HER2 was 17.3% (*n* = 45, *p* = 0.031), most driven by cases switching to or from HER2-low expression. In 18 of the cases (5.0%), HER2 status had changed from HER2-0 in primary tumor to HER2-low in metastatic lesions (23.1% of the HER2-0 primary breast cancer cohort), and in 17 of the cases (4.7%), HER2 status had changed from HER2-low to HER2-0 (13.7% of the HER2-low primary breast cancer cohort). Ki67 changed from low in the primary tumor to high status in LNM for 22 patients (6.1%), while changes from high to low were observed in 40 patients (11.2%). In total, the subtype of the LNM was different in 34 cases (9.5%) compared with paired primary breast cancer (Fig. 3).

**Table 2:** Discordance in biomarker status between the primary breast cancer and synchronous axillary LNM

	Breast cancer (*n* = 358)	Lymph node (*n* = 358)	Discordance rate (%)	*κ*	*p*-value^*^
ER					
Negative	145 (40.5)	142 (39.7)	5.9	0.877	0.663
Positive	213 (59.5)	216 (60.3)			
PR					
Negative	190 (53.1)	205 (57.3)	8.7	0.825	0.012
Positive	168 (46.9)	153 (42.7)			
HER2					
HER2-0	78 (21.8)	76 (21.2)	12.6	0.8445	0.018
HER2-low	124 (34.6)	134 (37.4)			
HER2-positive	156 (43.6)	148 (41.3)			
Ki67					
< 30%	63 (17.6)	81 (22.6)	17.3	0.4632	0.031
≥ 30%	295 (82.4)	277 (77.4)			

^*^McNemar-Bowker test

*ER* estrogen receptor, *PR* progesterone receptor, *HER2* human epidermal growth factor 2

### Analysis of factors associated with biomarker discordance

Clinical and pathological factors were tested to search for correlation with the biomarker alterations (Table 3). The discordance rate of HR and HER2 appeared similar across different biopsy method of lymph node. HR discordance was observed more frequently when the ER status (*p* = 0.023) or PR status (*p* = 0.010) of primary tumor was negative, while HER2 discordance seemed to be more frequent when the HER2 status of primary tumor was HER2-0 or HER2-low (*p* < 0.001). Tumor stage, histological grade and histological type had no correlation with receptors conversion.

**Table 3:** Univariate analysis of factors associated with biomarker discordance

Characteristic	HR status			HER2 status			Ki67 status		
	Con	Dis	*p*-value	Con	Dis	*p*-value	Con	Dis	*p*-value
Age at diagnosis									
≤ 50	162	13	0.908	154	21	0.874	144	31	0.957
> 50	171	12		159	24		152	31	
Menopausal status									
Pre/perimenopausal	172	14	0.832	165	21	0.549	154	32	1
Postmenopausal	161	11		148	24		142	30	
Family history of breast cancer									
Yes	22	5	0.04	23	4	0.949	21	6	0.663
No	311	20		290	41		275	56	
Histological type									
Invasive ductal	296	23	0.881	279	40	1	265	54	0.738
Other	37	2		34	5		31	8	
Histological grade									
G1/G2	168	10	0.39	158	20	0.497	140	38	0.055
G3	161	15		151	25		153	23	
Clinical T stage									
cT1	61	3	0.126	53	11	0.365	51	13	0.487
cT2	216	21		208	29		195	42	
cT3-4	56	1		52	5		50	7	
Clinical N stage									
cN0	30	0	0.249	27	3	0.876	23	7	0.511
cN +	303	25		286	42		273	55	
ER status of PBC									
Negative	129	16	0.023	128	17	0.814	128	17	0.03
Positive	204	9		185	28		168	45	
PR status of PBC									
Negative	170	20	0.01	169	21	0.447	165	25	0.038
Positive	163	5		144	24		131	37	
HER2 status of PBC									
HER2-0	71	7	0.276	59	19	< 0.001	64	14	0.959
HER2-low	119	5		107	17		102	22	
HER2-positive	143	13		147	9		130	26	
Ki67 status of PBC									
< 30%	60	3	0.624	50	13	0.055	41	22	< 0.001
≥ 30%	273	22		263	32		255	40	
Biopsy method of lymph node									
CNB	293	23	0.78	276	40	1	267	49	0.023
SLNB	40	2		37	5		29	13	

*Con* concordance, *Dis* discordance, *HR* hormone receptor, *HER2* human epidermal growth factor 2, *ER* estrogen receptor, *PR* progesterone receptor, *PBC* primary breast cancer, *CNB* core needle biopsy, *SLNB* sentinel lymph node biopsy

In univariate analysis, positive ER status of primary tumor (21.1% vs. 11.7%, *p* = 0.030), positive PR status of primary tumor (22.0% vs. 13.2%, *p* = 0.038), low Ki67 expression (34.9% vs. 13.6, *p* < 0.001), and the application of SLNB (31.0% vs. 15.5%, *p* = 0.023), showed association with the change of Ki67 expression between primary breast cancer and axillary LNM. In multivariate analysis, low Ki67 expression of primary tumor (OR 3.15[1.67–5.90], *p* < 0.001) and the application of SLNB (OR 2.40[1.11–5.01], *p* = 0.022), remained independent factors that were predictive of the alteration in Ki67 expression.

### Exploratory analysis of neoadjuvant therapy response

**Fig. 4:**
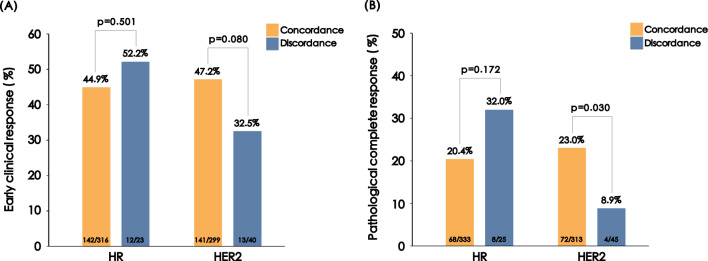
The influence of biomarker conversion on neoadjuvant therapy response. **A** Early clinical response. **B** Pathological complete response. *HR* hormone receptor, *HER2* human epidermal growth factor 2

The evaluation of pCR was conducted on a total of 358 patients, while 19 patients had missing assessments of early clinical response. Exploratory analysis did not reveal any statistically significant difference in neither pathological nor early clinical response to neoadjuvant therapy between the concordant and discordant groups for HR status (Fig. 4). Pathological complete response in concordant vs. discordant groups for HER2 status was observed in 72 of 313 patients vs. 4 of 45 patients (23.0% vs. 8.9%, *p* = 0.030, Fig. 4). However, no significant difference in early clinical response was observed according to the concordance of HER2 status (47.2% vs. 32.5%, *p* = 0.080, Fig. 4).

**Fig. 5:**
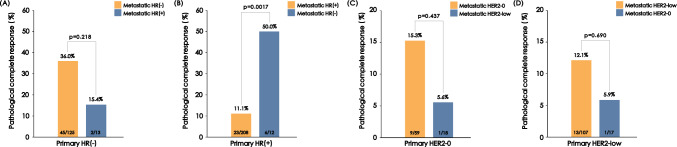
The influence of biomarker conversion on neoadjuvant therapy response according to primary tumor subtypes. **A** pCR in HR-negative primary breast cancer. **B** pCR in HR-positive primary breast cancer. **C** pCR in HER-0 primary breast cancer. **D** pCR in HER2-low primary breast cancer. *HR* hormone receptor, *HER2* human epidermal growth factor 2

In addition, the subgroup analysis was performed considering the impact of the receptor status in primary breast cancer. Among patients with HR-negative primary tumor, gain of HR-positivity (negative to positive) was associated with a tendency to have lower pCR rate, although the difference between groups was not statistically significant (36.0% vs. 15.4%, *p* = 0.218, Fig. 5). A significantly increased pCR rate was found in patients with loss of HR-positivity (positive to negative), compared to those with stable positive HR expression (50% vs. 11.1%, *p* = 0.0017, Fig. 5). When evaluating the potential impact of HER2 expression change in HER2-low cases on the neoadjuvant therapy response, there was no pCR difference for concordant HER2-0 vs. gain of HER2-low expression (15.3% vs. 5.6%, *p* = 0.437, Fig. 5) or concordant HER2-low vs. loss of HER2-low expression (12.1% vs. 5.9%, *p* = 0.690, Fig. 5).

The evolution of HER2 expression from baseline biopsy to residual disease after neoadjuvant therapy in patients failing to achieve pCR was analyzed. In particular, among the 17 non-pCR cases with HER2-0 primary tumor and HER2-low axillary LNM, more than a third exhibited HER2-low residual disease which consistent with axillary LNM (Supplementary Table 2).

## Discussion

The instability of ER, PR, HER2, and Ki67 expression during the progression of breast cancer have been widely reported, but the primary focus was on postoperative locoregional recurrence or distant metastasis. In our retrospective cohort of 358 breast cancer patients undergoing neoadjuvant treatment, evolution of biomarker expression from primary tumor to synchronous axillary LNM was explored using baseline biopsy specimens instead of the tumor tissue obtained from radical surgery. Change of ER, PR, HER2, and Ki67 expression was observed in 5.9%, 8.7%, 12.6%, and 17.3% of patients, respectively. Therefore, ER appeared to be most stable receptor, whereas HER2-low expression is highly unstable during disease evolution. Our findings demonstrated instability in clinically used markers throughout tumor progression in early stage, which eliminated the impact of selective pressure induced by anti-tumor treatment.

Compared to the similar studies, the discordance rate of HR was relatively low in our cohort, while changes of HER2 statues in axillary LNM were perceived more frequently. Various discordance rates reported were 1.1–28.8%, 10.6–31.7%, and 2.9–15.9% for ER, PR, and HER2, respectively (Ba et al. 2014; Falck et al. 2010; Janeva et al. 2023; Kinoe et al. 2018; Li et al. 2016; Sujarittanakarn et al. 2020; Weydandt et al. 2022; Zhao et al. 2015). This wide variability of the concordance rates can be associated with the heterogeneity in patient characteristics, biopsy method, or cut-off values for biomarkers. All patients in analysis received neoadjuvant therapy, meaning a higher proportion of HER2-positive and triple-negative subtype in our cohort. And the present study firstly adopted the HER2-low as a distinct category in analyzing receptors discordance between primary breast cancer and synchronous axillary LNM, which may result in elevated discordance rate of HER2. Especially, several previous studies included axillary lymph node specimens that were surgically removed after systemic treatment (Weydandt et al. 2022; Zhao et al. 2015).

Tissue sampling and assay method may be one of explanations for the difference between primary breast cancer and paired metastases in lymph node. Although adequate specimens were obtained from ultrasound-guided CNB and the biopsy method of axillary lymph node was not a predictive factor for ER, PR, and HER2 conversion in our study, the limitations of needle biopsy should not be disregarded (Chen et al. 2012). To minimize the variation potentially caused by assay techniques, the cases included in our study were limited within the past ten years and all assays in our laboratory were performed following proper guidelines. Given that IHC is observer-dependent and semiquantitative, the possibility to perform mRNA testing by real-time quantitative polymerase chain reaction (qPCR) as alternative to IHC to reduce the variability deserves further investigation (Marchio et al. 2021). In clinical practice, however, IHC remains the gold standard for diagnostic purposes.

Biomarker conversion in metastatic breast cancer should be seen more as a true biological phenomenon and not solely the result of limited accuracy of technique. It has been shown that primary breast cancers demonstrate intra-tumor heterogeneity with genetically distinct cellular populations formation as they evolve (Dentro et al. 2021). Genetic changes and phenotypic plasticity may also be involved in subclonal expansion and spread of disseminated tumor cells (DTCs) with various invasion ability. Adapting the epigenetic, transcriptional, and post-transcriptional landscapes is required for metastatic cells to undergo each step of the metastatic cascade (Gerstberger et al. 2023). Actually, breast cancer tends to preferentially relapse in certain organs although DTCs can spread to virtually all organs. Each organ varies in vascular and nutrient supply, immune microenvironment, and stromal composition, thus brings difference influence for infiltrating cancer cells (Obenauf and Massague 2015). This view is supported by the different receptors conversion rates among different metastatic sites and the discordance in receptor profiles among metastatic sites in cases with multi-organ metastasis (Schrijver et al. 2018). Tumor draining lymph node, especially, has been demonstrated as a critical site for inducing tumor immunosuppression. Overall, intrinsic cancer cell traits and composition of host-organ microenvironments together form the metastatic tumors with characteristics different from those of the primary.

The clinical implication of this instability in locally advanced breast cancer is important, whereas gain of HER2 and HR generally means sensitivity to trastuzumab/pertuzumab and endocrine therapy, respectively. In our cohort, the ER positive rates did not show any significant difference between primary tumors and paired axillary LNMs (*p* = 0.663). In line with other studies, a change of PR was observed more frequently than an ER change in our study, as well as changes of positive to negative PR (6.4%) *versus* negative to positive (2.2%) were perceived more often (Dieci et al. 2013; Liedtke et al. 2009; Shiino et al. 2016). As an ER-dependent gene product, PR loss in breast cancer metastases without changes in ER status observed is of uncertain clinical significance, as ER is generally considered a stronger indicator of response to endocrine therapy. Our results suggested that patients showing HR conversion responded to neoadjuvant chemotherapy better than patients who remained ER or PR positive in their axillary lymph node metastasis. Meanwhile, loss of HR expression was associated with a significant worse overall survival in previous studies focused on distant metastasis (Dieci et al. 2013; Lindstrom et al. 2012). Overall, a change of HR status during the progression of breast cancer indicates specific biology features, rather than a fortuitous events. The relevance of this phenomenon may be more pronounced in the context of neoadjuvant endocrine therapy, but further research is needed to answer this question.

The evolution of HER2 expression from primary breast cancer to axillary LNMs deserves more attention because of the introduction of the newly proposed HER2-low category. In our study, the overall rate of HER2 discordance was 12.6%, mostly represented by HER2-0 switching to HER2-low (5.0%) and HER2-low switching to HER2-0 (4.7%). Such high instability of HER2-low expression during disease evolution has been reported in advanced disease stage (Bergeron et al. 2023; Miglietta et al. 2021). Despite the hypothesis that the complex crosstalk between HR and HER2 pathways, HR status of primary tumor did not show any association with HER2 heterogeneity. A significantly decrease in pCR rate was observed in patients with unstable HER2 status, but not in the HER2-0/HER2-low subgroup. The possible explanation for such observation is that the instability was mostly driven by cases with HER2-0/HER2-low phenotype, which is known to be not appropriate for trastuzumab/pertuzumab. Actually, HER2-low status is not an independent predictor of pCR in current neoadjuvant setting, while a limited number of cases experience the conversion from HER2-negative to HER2-positive (Li et al. 2023). So, it is appropriate to determine the administration of neoadjuvant anti-HER2 targeted therapy based on the HER2 status of the primary tumor at present stage. However, the use of anti-HER2 ADCs in the treatment of early HER2-low breast cancer is still far from implementation in the clinical practice, which may obscure the clinical implication of evolution of HER2-low in locally advanced breast cancer.

It should be noted that the ASCO/CAP guideline recommendations were optimized to identify HER2-positive tumors (Anderson et al. 2023). Before the development of ADCs, HER2 IHC 0 and 1 + scores were both considered to be HER2-negative, and discriminating between the two had no clinical implications. So, it is not surprising that the scoring accuracy for HER2 IHC in the low range (0 and 1 +) is suboptimal (Fernandez et al. 2022). The research findings related to HER2-low should be reviewed with caution before new clinical applicable assays to reproducibly identify HER2-low tumors is provided.

Our study presented the largest cohort to provide additional date on receptor conversion in axillary lymph node metastasis of breast cancer, and investigate the predictive value of this instability on neoadjuvant treatment for the first time. However, this study has the following limitations. First, this was a single-institution retrospective study based on medical records. Second, the sample size was considered to be relatively low because of the low discordant rate, although many of the studies discussing this topic had cohorts with less than 200 patients. Third, we excluded ninety-four patients with unavailable ER, PR, HER2, or Ki67 status, which may introduce selection bias. However, most of these patients were diagnosed by fine needle aspiration (FNB), and the specimens were actually insufficient to preform accurate IHC. Another limitation of the present study is the lack of long-term prognostic information and molecular data (e.g., PAM50), which is a topic with worthy of researching farther.

## Conclusion

Receptor discordance between primary tumor and synchronous axillary LNM appears to already exist before any anti-tumor therapy, which might reflect intra-tumor heterogeneity and phenotypic plasticity of breast cancer. The instability of ER, PR, and HER2 expression has limited clinical impact on neoadjuvant therapy at current stage, but further investigation is warranted with the incremental application of endocrine drugs and ADCs in neoadjuvant therapy.

## Supplementary Information

Below is the link to the electronic supplementary material.

Supplementary file1 (DOCX 15 KB)

## Data Availability

All data generated or analyzed during this study are included in this published article and its supplementary information files.
