# Supplementary material for: Biomarker conversion from primary breast cancer to synchronous axillary lymph node metastasis and neoadjuvant therapy response: a single-center analysis
Source: J Cancer Res Clin Oncol. 2024 Jun 8;150(6):297. doi: 10.1007/s00432-024-05834-y (PMC11162378; doi:10.1007/s00432-024-05834-y)
Supplement: Supplementary file 1 — Supplementary file1 (DOCX 15 KB) [file 432_2024_5834_MOESM1_ESM.docx]

**Supplementary Table 1.** Antibodies and automated IHC platform used for tissue staining.

| **Biomarker** | **Antibody** | **Automated IHC Stainers** |
| --- | --- | --- |
| ER | 1D5, DAKO | DAKO Autostainer Link 48 |
| PR | 636, DAKO | DAKO Autostainer Link 48 |
| HER2^1^ | 4B5, VENTANA | VENTANA BenchMark GX |
| Ki67 | MIB1, DAKO | DAKO Autostainer Link 48 |

^1^ Samples with IHC score 2+ for HER2 were additionally assessed using HER2 gene test kit (FISH; Guangzhou Anbiping Medicine Technology Co., Ltd.).

ER, estrogen receptor; PR, progesterone receptor; HER2, human epidermal growth factor 2.

**Supplementary Table 2.** Receptors evolution from baseline biopsy to residual disease after neoadjuvant therapy in primary HER2-0 cases.

| **No.** | **Primary tumor** | **Synchronous axillary LNM** | **Residual disease** |
| --- | --- | --- | --- |
| 3 | HR+/HER2-0 | HR+/HER2-low | HR+/HER2-0 |
| 25 | HR+/HER2-0 | HR+/HER2-low | HR+/HER2-0 |
| 28 | HR+/HER2-0 | HR+/HER2-low | HR-/HER2-0 |
| 32 | HR-/HER2-0 | HR-/HER2-low | HR-/HER2-0 |
| 36 | HR+/HER2-0 | HR-/HER2-low | HR-/HER2-0 |
| 51 | HR+/HER2-0 | HR+/HER2-low | HR+/HER2-low |
| 58 | HR-/HER2-0 | HR-/HER2-low | HR-/HER2-0 |
| 103 | HR+/HER2-0 | HR-/HER2-low | HR-/HER2-0 |
| 106 | HR-/HER2-0 | HR-/HER2-low | HR-/HER2-0 |
| 154 | HR-/HER2-0 | HR-/HER2-low | HR-/HER2-low |
| 202 | HR+/HER2-0 | HR+/HER2-low | HR+/HER2-0 |
| 234 | HR+/HER2-0 | HR+/HER2-low | HR+/HER2-low |
| 257 | HR-/HER2-0 | HR-/HER2-low | HR+/HER2-low |
| 260 | HR+/HER2-0 | HR+/HER2-low | HR+/HER2-low |
| 272 | HR+/HER2-0 | HR+/HER2-low | HR+/HER2-0 |
| 297 | HR+/HER2-0 | HR+/HER2-low | HR+/HER2-low |
| 340 | HR+/HER2-0 | HR+/HER2-low | HR+/HER2-0 |

LNM, lymph node metastasis; HR, hormone receptor; HER2, human epidermal growth factor 2.
